# First person – Claudia Collier

**DOI:** 10.1242/bio.062061

**Published:** 2025-06-06

## Abstract

First Person is a series of interviews with the first authors of a selection of papers published in Biology Open, helping researchers promote themselves alongside their papers. Claudia Collier is first author on ‘ Persistent enteric neuroinflammation chronically impairs colonic motility in a pyridostigmine bromide-induced mouse model of Gulf War illness’, published in BiO. Claudia is a PhD candidate in the lab of Dr Shreya Raghavan at Texas A&M University, College Station, TX, USA, interested in the intersection of using tissue engineering models to investigate neuro-immune interactions.


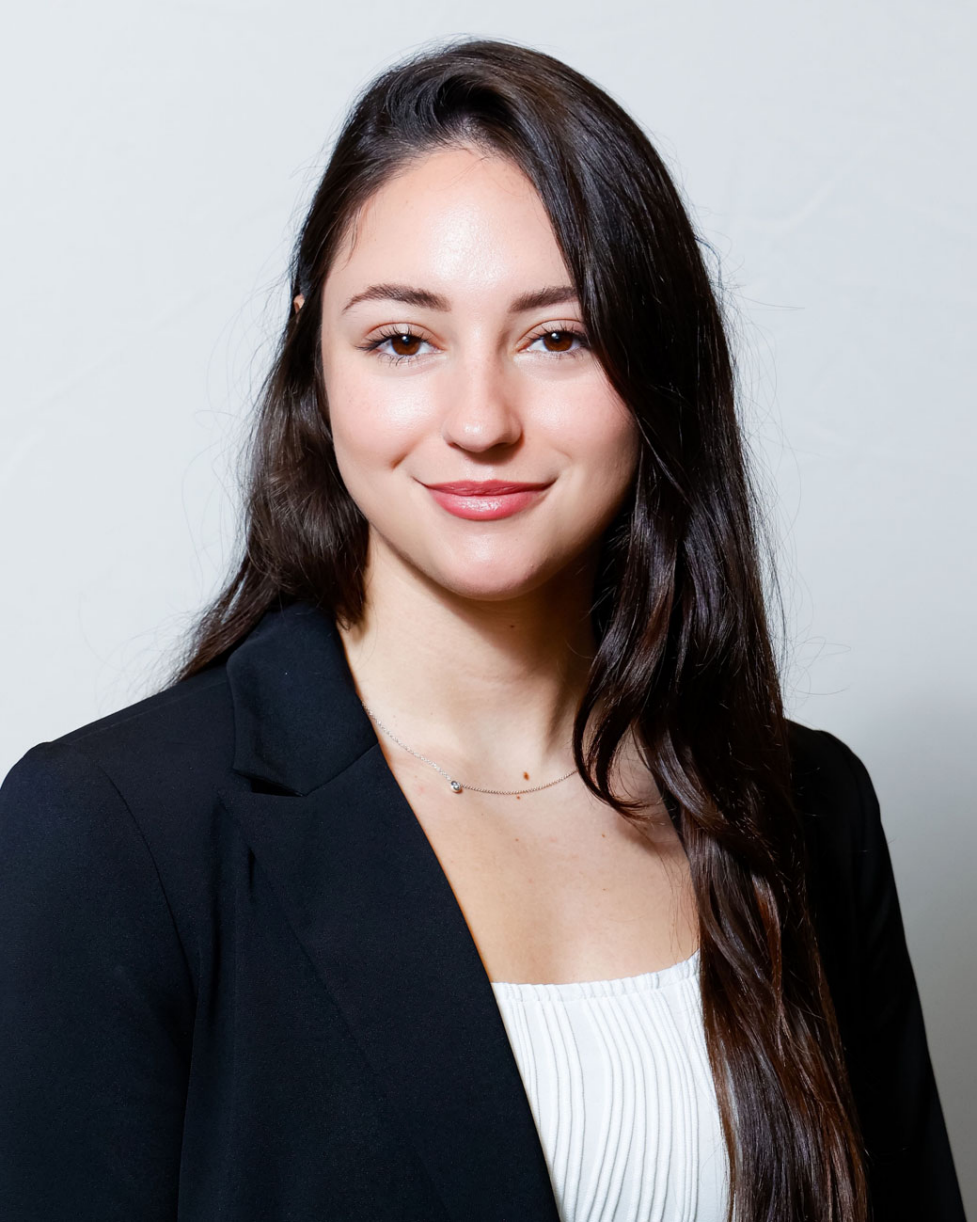


**Claudia Collier**

**Describe your scientific journey and your current research focus**

I've always been fascinated by the body's ability to heal, and early on I envisioned becoming a medical doctor. That changed when I joined my first research lab as an undergraduate and studied how addiction alters the central nervous system. I became captivated by the potential of research to uncover biological mechanisms and drive real therapeutic advances. This led me to explore biomedical engineering, where I worked on nanotechnology-based approaches for neuromodulation in Parkinson's disease. Eventually, I found a PhD opportunity that brought all my interests together – neuroscience, immunology, and engineering – through the study of how inflammation impacts the nervous system of the gut. I am now a PhD candidate in Dr Shreya Raghavan's lab at Texas A&M University, where I investigate enteric neuroinflammation in the context of Gulf War illness (GWI) – a condition that also personally motivates me, as my mother is a Gulf War veteran living with its effects. My research integrates animal models, *in vitro* cell systems, and 3D bioengineered colon constructs to examine how toxic exposures cause lasting changes to gut motility, neural signalling, and immune activation. By combining these platforms, I aim to uncover mechanistic and functional changes in the enteric nervous system and develop systems that can be used for future therapeutic screening. This work has been both scientifically fulfilling and deeply meaningful, as it brings me closer to understanding and addressing a condition that affects many veterans and their families.

**Who or what inspired you to become a scientist?**

One of the most influential figures in my scientific journey was my summer research mentor at the University of Connecticut, Dr Alexander Jackson. During my REU experience, he not only introduced me to the excitement of neuroscience research, but also completely reframed what science could look like. His patient mentorship, genuine curiosity, and storytelling approach to science made research feel alive and personal. He showed me that being a scientist wasn't just about collecting data – it was about asking thoughtful questions, staying curious, and communicating discoveries in a way that could inspire others.

**How would you explain the main finding of your paper?**

We discovered that exposure to a chemical linked to Gulf War illness can change how the colon moves by affecting the nerves and immune cells that help control gut function. Our study showed that even after removing the chemical, these changes don't fully go away suggesting that the damage to the gut's ‘brain’ may be long-lasting losing the ability to repair itself.

even a brief, one-time toxic exposure can cause long-lasting nerve damage and inflammation in the gut

**What are the potential implications of this finding for your field of research?**

Our findings shed light on how even a brief, one-time toxic exposure can cause long-lasting nerve damage and inflammation in the gut. This helps explain why many Gulf War veterans continue to suffer from chronic digestive issues decades after exposure. By uncovering how enteric nerve damage and inflammation persist in the colon, our work opens the door to developing therapies that target the underlying biology of gut dysfunction – not just in Gulf War illness, but potentially in other chronic gastrointestinal conditions where neuro-immune balance is disrupted​.

**Figure:**
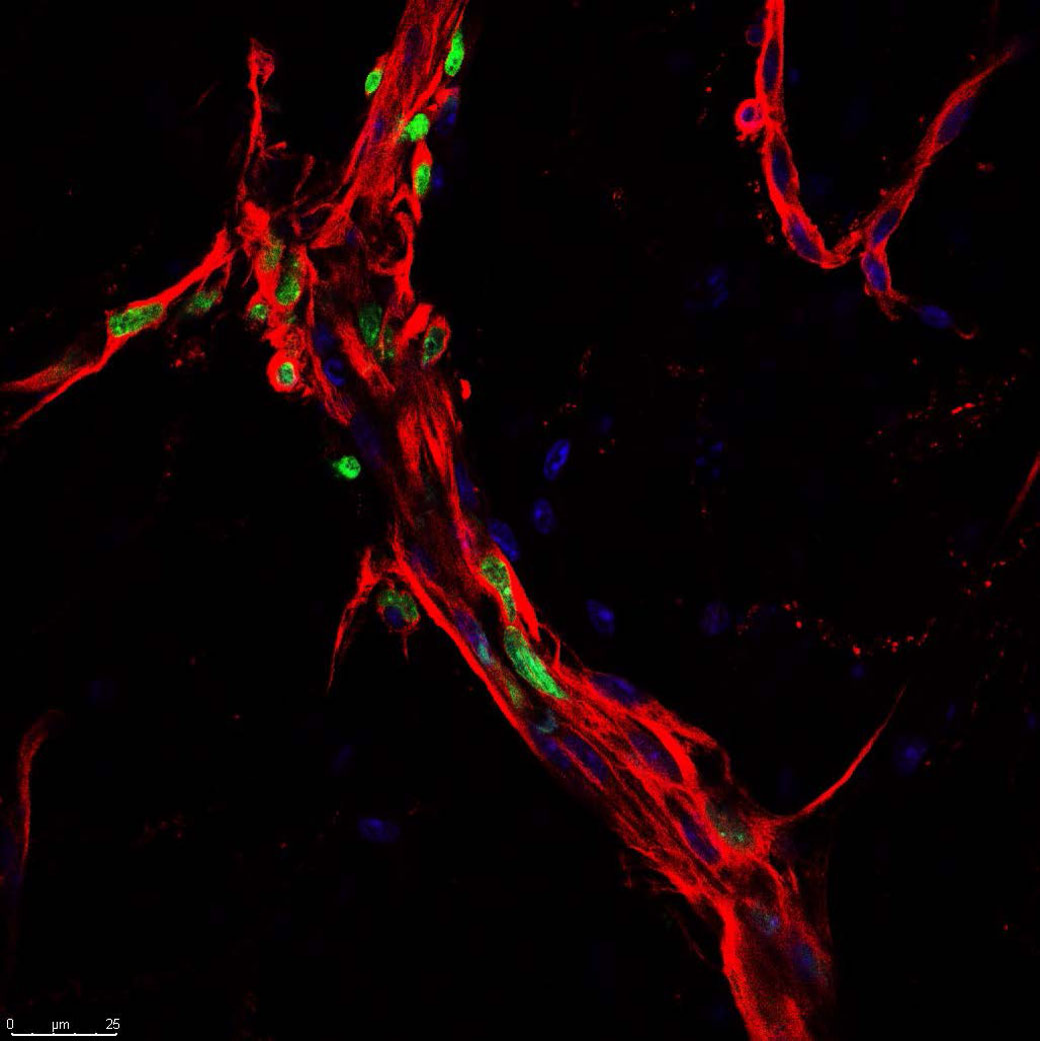
Proliferating cells (Edu, green) within the myenteric plexus (βIII Tubulin, red).

**Which part of this research project was the most rewarding?**

The most rewarding part of this project was capturing the image shown in Fig. 4H, where I visualized an enteric neural stem cell (ENSC) surrounded by pro-inflammatory macrophages within the colonic tissue of PB-exposed mice. This moment was incredibly impactful – not just technically, but conceptually. It provided visual evidence that inflammation may be disrupting the very cells responsible for repairing the enteric nervous system. That single image fundamentally shifted how I thought about gut regeneration under chronic inflammatory conditions and reinforced the importance of neuro-immune crosstalk in long-term gut dysfunction. Seeing something so central to our hypothesis unfold in a single field of view was both affirming and motivating.

**What do you enjoy most about being an early-career researcher?**

I love the freedom to be creative in our lab and I love that I am also able to work on multiple projects. It has been extremely satisfying to build experiments from scratch, ask questions, and then be able to tell a story.

I love the freedom to be creative in our lab

**What piece of advice would you give to the next generation of researchers?**

Don't be afraid to fail. Some of the most important learning happens when things don't go as planned – what matters is how you adapt and keep moving forward. Also, find mentors and peers who uplift you; research is a team effort.

**What's next for you?**

I will be defending my PhD in August and graduating in December. After that, I plan to transition into a career at the intersection of science and technology, biotech! I'm excited to explore roles that bridge scientific discovery with real-world impact.

**What personal connection do you have to your research, and how has it shaped your perspective as a scientist?**

My work on Gulf War illness is deeply personal – my mother is a Gulf War veteran who lives with many of the long-term symptoms that we now believe stem from toxic exposures. Having this personal connection has shaped the way I approach science. It's made me more aware of the people behind the data and more committed to translating research into something that can genuinely help others. It's also given me a unique perspective on how science, policy, and lived experience intersect, which continues to motivate my future path in public health-oriented research.
